# Congenital hearing loss: introduction of the R67 large gene panel in England

**DOI:** 10.1017/S0022215124001348

**Published:** 2025-01

**Authors:** Ahmed Fazili, Hannah Blanchford, Kostas Tsioulos

**Affiliations:** 1 St George's University of London, London, UK; 2 Department of Audiovestibular Medicine, St George's Hospital, London, UK

**Keywords:** diagnostic techniques, genetics, hearing loss, inner ear, otological, sensorineural hearing loss

## Abstract

**Background:**

Congenital hearing loss is a chronic condition which occurs worldwide. In the past, investigations focused on testing the most common genes associated with hearing loss (such as Connexin 26-related hearing loss). Targeted testing of specific genes was requested only when a particular syndrome was suspected. Recent advances have led to the development of a large gene panel which utilises next-generation sequencing to simultaneously test for pathogenic variants in many genes associated with hearing loss.

**Aim:**

This review article aims to highlight the changes in the approach to congenital hearing loss in the context of the R67 gene panel, and how its use may increase the efficiency of the diagnosis and management of this condition.

**Conclusion:**

The use of this large gene panel has revolutionised the approach to hearing loss. Uptake of this large gene panel has resulted in prompter diagnosis and therefore more appropriate clinical management.

## Introduction

**Figure 1.**
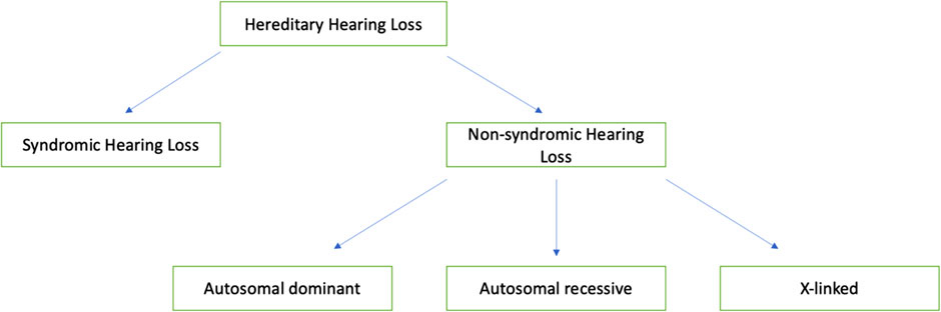
Causes of congenital hearing loss.

Congenital hearing loss can be categorised as syndromic or non-syndromic (Figure 1).^1^ Fifty to eighty per cent of congenital hearing loss has a genetic aetiology. Approximately 70 per cent of people with a genetic cause of congenital hearing loss have ‘non-syndromic sensorineural hearing loss’.^1,2^ Studies have shown that hearing loss affects up to 10 million people in the UK and that 45,000 of those are children.^3^

**Figure 2.**
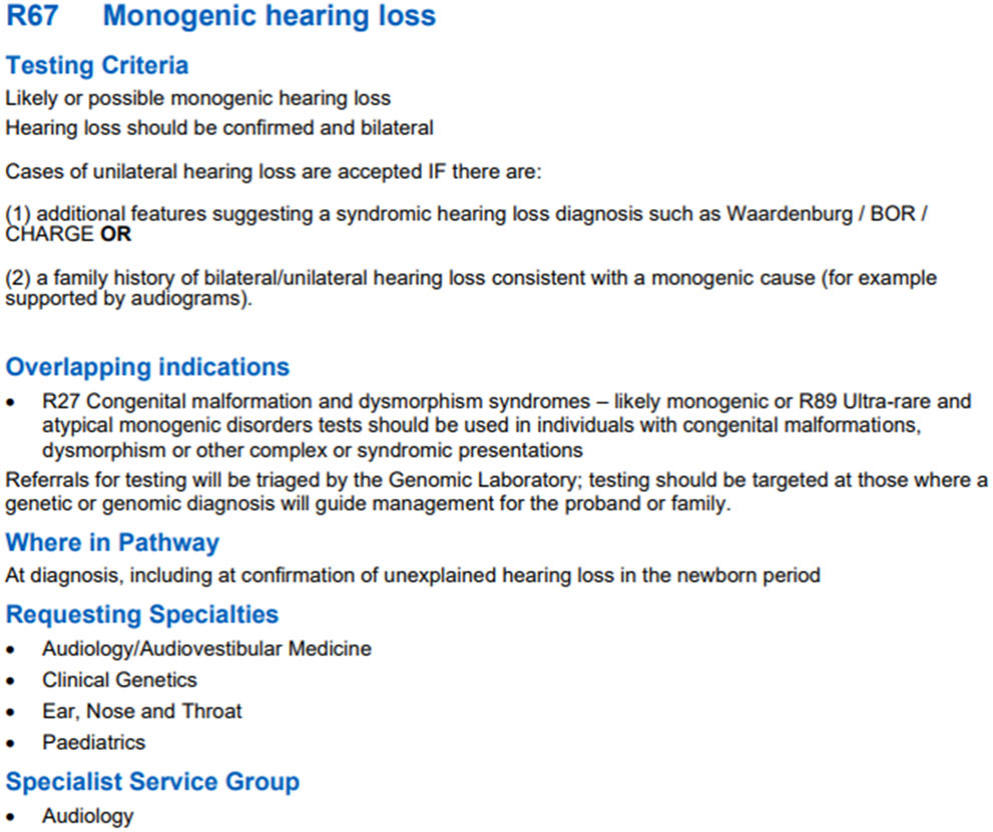
National Genomic Test Directory testing criteria for rare and inherited disease, October 2021 v2, p. 161 (https://www.england.nhs.uk/wp-content/uploads/2018/08/rare-and-inherited-disease-eligibility-criteria-v2.pdf).

Given the high prevalence of hearing loss in the UK, the newborn hearing screening programme was introduced in 2001, to ensure early identification of hearing loss in newborns,^4^ with a study analysing the effectiveness of the programme concluding that the programme delivered satisfactory outcomes in terms of age of referral, identification, and intervention of hearing loss through screening of 169,487 infants.^5^ Since its conception, the R67 panel (Figure 2) (‘R’ for ‘rare and inherited disease’ and ‘67’ for the number at which monogenetic hearing loss features in the National Genomic Test Directory) is regularly updated based on emerging evidence and review of previous evidence.

In 1997, researchers identified a large family in Pakistan where multiple members reported isolated cases of hearing loss.^6^ After genetic sequencing, it was revealed that all affected individuals shared a genetic mutation in a single gene, GJB2. The GJB2 gene codes for protein Connexin-26 which is thought to be essential for hearing. Given the lack of knowledge of the human genome and limitations in techniques at the time, very few genes were known to cause congenital hearing loss, leading many doctors to believe that GJB2 and GJB6, both coding for the same molecular protein, were two of very few mutations responsible for non-syndromic deafness. For this reason, Sanger sequencing of GJB2 and GJB6, followed by mitochondrial DNA 1555 G>A, were the mainstay of investigations in children with congenital bilateral severe to profound sensorineural hearing impairment in the absence of other clinical features,^2^ with further investigations for a limited number of genes if there were additional family history and/or any syndromic features. However, this approach left many without a genetic diagnosis.

More than 20 years on from the discovery of GJB2, studies such as the Human Genome Project of 2003 and the 100,000 Genomes Project of 2018 have advanced the technology for sequencing and increased our understanding of genes implicated in hearing loss. One of the legacies of the 100,000 Genomes Project in England has been the establishment of the NHS Genomic Medicine Service hearing loss panel.^7^ This advancement has not only revolutionised the genetic testing protocols for sensorineural deafness in children, but also allowed for early detection of syndromes that can inform more individualised management and can also have significant implications for patients and their families. This article focuses on the integration of panel testing into routine NHS practice, how it has become the mainstay of genetic investigation of congenital deafness, and its implications in the clinical context.

## Discussion: advances in genetics and genomics

### The role of the 100,000 Genomes Project

The 100,000 Genomes Project was established in 2012, with the aim of sequencing 100,000 whole genomes from around 85,000 NHS patients with a rare disease, their families and patients with cancer.^8^ The project utilised whole-genome sequencing to identify new genetic diagnoses for patients with rare inherited diseases and cancer. It aimed to provide a better understanding of diseases at the molecular genetic level. By understanding disease at a molecular level, it is hoped that novel and more effective treatments can be developed which specifically target the pathogenic process. In the context of congenital hearing loss, this project has allowed geneticists to identify many genes implicated in deafness, building on previous research into underlying genetic causes.

### Rare disease eligibility criteria

Rare disease eligibility criteria were created to give doctors, scientists and researchers an indication of which conditions were approved for recruitment in the 100,000 Genomes Project, ensuring that every patient included in the project provided new information not yet recorded. Under the broad title of ‘non-syndromic hearing loss,’ the criteria give specific guidelines for: congenital hearing impairment, auditory neuropathy spectrum disorder, and autosomal dominant deafness.^9^ In terms of congenital deafness and prior understanding, the guidelines strongly recommended genetic testing of GJB2 and SCL26A4 genes prior to inclusion in the project.

### Introduction of the Gene Testing Panel for sensorineural hearing loss

In the past, the high cost of genomic technologies and limitations in the Sanger sequencing method meant that, in cases of non-syndromic hearing impairment (in both children and adults), testing was limited to one gene at a time. As a result, large numbers of cases were left without a genetic diagnosis. Following on from the 100,000 Genomes Project, a deafness gene panel was created, allowing multiple genetic causes to be tested simultaneously.

Although referred to as a ‘non-syndromic hearing loss panel,’ the R67 Gene Testing Panel consists of 135 genes which are known to be associated with syndromic and non-syndromic hearing loss. This testing panel was created over time through the work of the 100,000 Genome Project and by collaborating with health professionals that submitted clinical evidence for certain genetic entities associated with hearing loss.

The currently available panel undergoes regular review by the Genomics Medical Service. Every gene that is suggested to be part of the panel is given a green, amber or red status depending on the current evidence level, with green-level genes being selected for the panel once this process is complete. It is therefore the presence and structure of these green-level genes in the patient's DNA that are the centre of the Gene Testing Panel investigation.

As of February 2021, the success of this deafness gene panel has prompted the NHS to include it in the standard practice of testing congenital hearing loss. In the past, individual departments were invoiced the cost of every patient referred for the deafness gene panel, which stood at £1030 for NHS patients and £1380 for private patients. However, since adoption of the service by the NHS, funding for genomic investigations has been centralised to the laboratory and testing is available for those that meet the eligibility criteria.

### Requesting the deafness gene panel

Before using the panel, doctors must first confirm whether their patient is eligible for this service. Requesting clinicians can be audiovestibular physicians, ENT specialists, paediatricians or clinical geneticists. The National Genomic Test Directory sets out clear guidelines that must be met by patients to use the Gene Testing Panel.^10^ Patients with a possible monogenic hearing loss are considered for the R67 panel. In cases of confirmed bilateral hearing loss, the R67 is also immediately considered a useful investigative tool. However, cases of unilateral hearing loss may be accepted if there are features suggestive of a syndromic hearing loss, or a family history of bilateral and/or unilateral hearing loss consistent with a monogenic cause. If the criteria are met, the R67 can be considered for both adults and children, regardless of age, provided it has clinical utility (e.g. in the prediction of hearing loss progression, involvement of other systems or organs that can be proactively investigated, or for family planning).

### How the test is performed

Once use of the tool is deemed acceptable by the leading clinician, the basic wet-lab workflow remains the same: obtaining the DNA sample, library preparation and finally sequencing. Library preparation is the first step in next-generation sequencing and allows for the target genes to be sequenced through adherence to the sequencing flowcell.^11^ In the context of the Gene Testing Panel, different institutions use different forms of library preparation. This varies from targeted panel capture, in which only the genes in the panel are sequenced, to whole genome sequencing, where the entirety of genes in the DNA sample are sequenced, with the geneticists then focusing on particular genes of interest.

Once the patient's DNA has been correctly sequenced, it is then compared to the reference DNA template generated by the Human Genome Project 2003. The Gene Testing Panel algorithm then highlights the changes in sequence, and the American College of Medical Genetics and Genomics (ACMG) Variant Classification Guidelines^12^ are used to determine whether this variant has a clinically significant effect on the phenotype.

The R67 panel utilises an initial subpanel analysis. As a result, a set of nationally agreed genes are the first focus, meaning the panel primarily either eliminates or confirms whether the hearing loss phenotype has occurred as a result of a frequent mutation. It is only in cases where this subpanel is found to be negative where the entire panel is then analysed. The panel testing process ceases if a pathogenic variant is found in one of the subpanel genes analysed and if this variant matches the phenotype of the patient.

In terms of the turnaround time from receiving the sample to official report, the current target, as for all genetic tests set nationally, stands at 84 calendar days. It is also worth noting that, although the R67 panel is available only in England, other countries have adopted similar gene panels for hearing loss that reflect their local populations and resources.

### ACMG variant classification guidelines

Each human genome has 3–4 million genomic variants compared to the reference genome, with only a minority of these being causative of a monogenic disease. In 2015, the American College of Medical Genetics and Genomics and the Association for Molecular Pathology published criteria to classify the pathogenicity of a specific variant.^14^ The ACMG guidelines were adopted by UK genetic diagnostic laboratories on 11 November 2016. These guidelines establish a standard classification system for variants based on their likelihood of contributing to a disease phenotype: ‘pathogenic,’ ‘likely pathogenic,’ ‘variant of uncertain significance,’ ‘likely benign’ or ‘benign.’ Alongside key information concerning the gene, its protein structure, and disease mechanism, accurate variant interpretation also requires detailed clinical history of the patient and family.

### Variants of uncertain significance

The presence of variants of uncertain significance requires careful interpretation by clinicians. It is often beneficial to have discussions with the wider multidisciplinary team for these complex findings so that their significance can be reviewed in the context of the family pedigree and clinical presentation. Re-evaluation of variants of uncertain significance may be prompted by new evidence, testing of affected family members or changes to a patient's phenotype.

A paper published in 2015 estimated that the likelihood of a variant of uncertain significance being pathogenic ranged from 10–90 per cent.^13^ Given the possibility that these variants may still have a link to the phenotype, an unofficial temperature gradient sub-classification system is used in the UK to further distinguish variants of uncertain significance’. Although the terms of this system are not typically included in the report seen by the patient, the use of words ‘hot variant of uncertain significance’, ‘tepid variant of uncertain significance’ or ‘cold variant of uncertain significance’ all denote the level of evidence currently supporting the variant being pathogenic.

According to the ACMG Variant Classification Guidelines, ‘hot variants of uncertain significance’ should be considered for reporting when there is a high level of supporting evidence, with any additional evidence resulting in re-classification to pathogenic. As a result, it is recommended that the summary of the report state ‘inconclusive result: consider further action,’ indicating that additional tests beyond the Gene Testing Panel may change the outcome.

Similarly, ‘tepid variants of uncertain significance’ refers to a moderate level of supporting evidence, and ‘cold variants of uncertain significance’ is used where there is a lower level than either ‘hot’ or ‘tepid.’ Given the present, albeit variable, probability that a variant of uncertain significance may be re-classified, it is imperative that variant data are stored for future re-analysis. Databases such as DECIPHER^14^ and ClinVar^15^ allow the sharing of variant data on a global scale, whilst ensuring security. Re-evaluation of a variant of uncertain significance may be prompted by release of new evidence, request for a family member test or progression of a patient's phenotype that raises questions on the original diagnosis.

### Advantages and disadvantages of the deafness gene panel^16^

One of the main advantages of the deafness gene panel is the simultaneous testing of multiple genes. This means the likelihood of discovering the cause of hearing loss is increased, whereas the focus on one specific gene per genetic test in the past resulted in missed or late diagnoses. Furthermore, analysis of multiple genes increases the probability of earlier identification of syndromic hearing loss.

Syndromic hearing loss refers to hearing loss in the context of a broader clinical condition, in which deafness is one of many symptoms. For example, those diagnosed with Usher syndrome often present with an isolated deafness, the use of the panel enables the identification of a pathogenic variant associated with Usher syndrome prior to the child developing visual impairment. Likewise, identification of a genetic condition causing long QT syndrome in a deaf child, can enable clinicians to treat the heart condition and prevent sudden death. Because the R67 panel only includes genes relating to hearing loss, there is no need to counsel parents or patients regarding the possibility of results indicating predisposition to, for example, cancers or neurodegenerative conditions.

Introduction of the deafness gene panel has brought with it certain challenges, especially to non-geneticists. The revelation of genetic changes in a patient opens questions as to whether other members of the patient's family could also be affected, resulting in anxiety. For this reason, the patient and their families must be counselled regarding the implications of genetic findings on the wider family, which is usually done as part of the consent process for the relevant tests.

Patients and their families should be made aware of the outcomes of panel testing. This includes finding a genetic change responsible for the phenotype, finding no relevant change, or finding a variant of uncertain significance. It is also possible that individuals are identified as ‘carriers’ for a recessive hearing-impairment gene, which can lead to further uncertainty and anxiety regarding reproductive risks.

- In the past, genetic investigation for cases of congenital hearing loss used Sanger sequencing to test for one or two of the most common genes associated with hearing loss
- In rare cases of syndromic hearing loss, the phenotype of the patient guided doctors and geneticists to pursue testing of specific genes
- Recent advancements in genetic techniques, the introduction of next-generation sequencing and the work of the 100,000 Genome Project has allowed simultaneous testing of multiple possible genetic causes of hearing loss
- This article focuses on how the Deafness Gene Panel, which consists of 135 genes known to be associated with syndromic and non-syndromic hearing loss, is used to form a diagnosis and management plan, and the way in which it has transformed the diagnostic approach to congenital hearing loss

Since its conception, the panel is regularly updated based on new, emerging evidence, and through the review of previous evidence. Although initially beneficial as it ensures clinicians are not limited by outdated research, the discovery of new evidence and genes for the future generation of patients does not consider patients in the past who have tested negative due to a lack of research on their genetic mutation. As there is no system in place currently to identify and re-assess these possible false-negative results, it is important to inform patients that they may wish to be re-reviewed, as more advanced testing could identify a genetic cause of their hearing loss in the future.

## Conclusion

The adoption of the congenital deafness gene panel by the NHS has changed the primary approach to hearing loss in children. Given the integration of this new investigative method into the management pathway of congenital deafness, specialists not only require easy access to the gene panel and the tests recommended for its use, but must acquire a new skill set to counsel patients and parents around the advantages and disadvantages of genetic testing and gain skills in interpreting and communicating the results. The primary role of genetic counselling resides with clinical geneticists. However, ENT specialists, audiovestibular physicians and paediatricians can acquire the skills to introduce the discussion surrounding the future implications of results found, so that the patient has a basic understanding once an onward referral to geneticists is made to delve deeper. Clinical scientists and clinical geneticists are able to support clinicians, and multidisciplinary teams are evolving to help with the integration of these new testing methods.

The ability to simultaneously test several possible aetiologies, coupled with the use of next-generation sequencing, has increased the efficiency of the investigative process compared to the past. Although the few drawbacks of the panel should not be ignored, the possibility of diagnosing complex syndromes hidden behind subtle clinical presentation presents an advantage over all other tests used in the past.
